# Failures of Cu-Cu Joints under Temperature Cycling Tests

**DOI:** 10.3390/ma15144944

**Published:** 2022-07-15

**Authors:** Po-Ning Hsu, Kai-Cheng Shie, Dinh-Phuc Tran, Nien-Ti Tsou, Chih Chen

**Affiliations:** 1 Department of Materials Science and Engineering, National Yang Ming Chiao Tung University, Hsinchu 30010, Taiwan; seraph8938@gmail.com (P.-N.H.); 911666alex@gmail.com (K.-C.S.); trandinhphuc1508@gmail.com (D.-P.T.); 2 Department of Materials Science and Engineering, National Chiao Tung University, Hsinchu 30010, Taiwan

**Keywords:** Cu-Cu joints, FEA models, stress gradients, interfacial voids, dielectrics

## Abstract

In this study, the failure mechanisms of Cu-Cu joints under thermal cycling were investigated. Two structures of dielectrics (PBO/underfill/PBO and SiO_2_) were employed to seal the joints. Stress gradients induced in the joints with the different dielectrics were simulated using a finite element method (FEM) and correlated with experimental observations. We found that interfacial voids were forced to move in the direction from high stress regions to low stress ones. The locations of migrated voids varied with the dielectric structures. Under thermal cycling, such voids were likely to move forward to the regions with a small stress change. They relocated and merged with their neighboring voids to lower the interfacial energy.

## 1. Introduction

With the current increasing demand for high performance computing (HPC) devices such as image sensors and central processing units (CPUs) [1], the traditional three-dimensional integrated circuit (3D IC) technology is facing considerable challenges [2,3]. One is the scaling-down of electronic devices. The direct bonding of metals can enable such an effort [4]. It is noted that the reliability of joints is greatly affected by bonding temperature and pressure. Pillar et al. [5] stated that the bonding can be stabilized using high-bonding temperature and pressure. Similar findings were also reported in the previous study [6]. Void density in the bonding can be suppressed by pre-bonding surface passivation [7]. Ide et al. [8] also proposed that Ag nanoparticles at the bonding interface can facilitate the Cu-Cu bonding. The crystal orientation of bonded metals also influences the bonding quality. Kief et al. [9] found that Cu with the same crystal orientation on both sides resulted in a good success rate for direct bonding. In particular, the <111> crystal orientation of Cu can greatly increase the success rate at a low bonding temperature [10]. The bonding quality can also be improved [11].

Although <111> crystal orientation is beneficial for direct bonding, a relatively high density of voids at the center of the bonding interface was still observed after a temperature cycling test without an applied force [12]. Various voids originally exist at metal surfaces and then move along grain boundaries to the center of the bonding interface due to stress gradients [13,14,15]. Moreover, previous studies [16,17] stated that stress gradient and residual stresses are not only induced by thermal cycling, but also the underfills (UFs) and/or dielectrics of joints. The effect of different UFs on the damage of micro ball grid array (microBGA) circuits during packaging was also reported [18]. Banijamali et al. [19] stated that the UF serves as a buffer layer, transferring the heat between substrate and microbump. A UF with a high glass transition temperature (*T*_g_) is recommended as it has a low Young’s modulus at a temperature above *T*_g_. Additionally, the shape of flip-chip solder joints also influences their viscoplastic strain energy density (SED) under thermal cycling and packaging of wafer-level chip-scale packages (WLCSPs). Previously, Darveaux [20] proposed an idea to predict the fatigue life of flip-chip solder joints. Such a prediction does not address complete factors due to a lack of information. However, these given viewpoints generally provide a feasible tool to predict fatigue life.

Large-scale Si interposers containing Cu-through-holes are commonly used in the through-silicon via (TSV) technologies. As replaced lead-free microbump TSV interposers with Si, the assembly yield of logic chips and organic substrates can be greatly improved. Si interposers can be employed to effectively suppress the formations of crack, delamination, and/or voids in the vicinity such as low-k, TSV, and joints [21]. For the assembly of wafer grades of 50 microns or less, the influence of capillary UF on the electrical functions of Cu pillars under thermomechanical stresses should be considered. The rounded-corner shape and coverage issues of Si-to-Si stacks can be also suppressed [22]. In this study, we aimed to investigate damage modes in Cu-Cu joints with different dielectrics under thermal cycling. Stress distribution in the joints was analyzed using ANSYS and then correlated with experimental observations.

## 2. Experimental and Simulation Section

**Figure 1:**
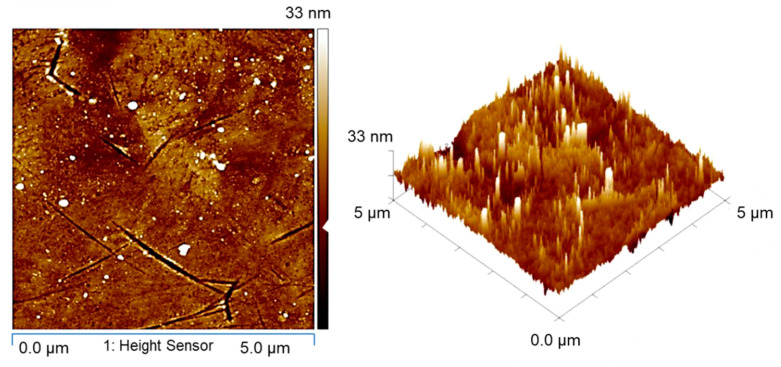
Typical AFM image of the Cu joints showing a roughness (*R*_q_) of ~3 nm.

**Figure 2:**
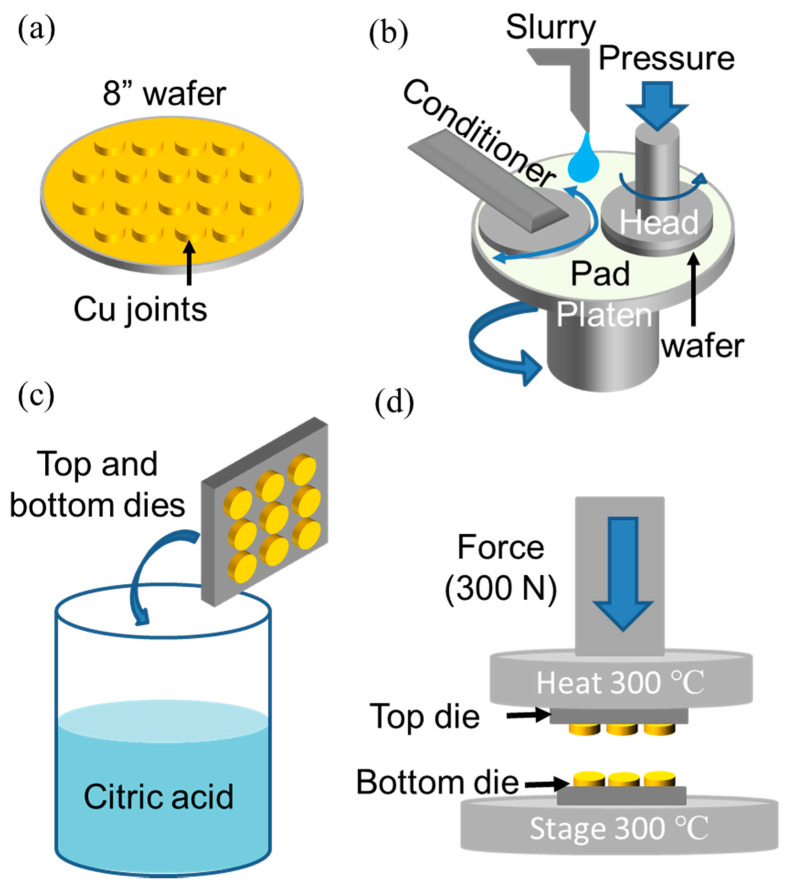
Schematic diagram of the Cu joint fabrication: (**a**) the DC electrodeposition, (**b**) CMP, (**c**) cleaning, and (**d**) bonding processes of the Cu joints.

In this study, various chips were designed and fabricated on an 8-inch patterned wafer. The wafers on the upper and lower sides were bonded and then cut into pieces. These diced wafers were employed to electroplate Cu redistribution lines (RDLs). All the RDLs were then covered by a passivation layer (photosensitive polybenzoxazole, PS-PBO) by spin coating. This was to prevent Cu layers from oxidation causing short circuits. The openings connecting Cu joints and RDLs were fabricated using photolithography. The Cu joints were electroplated in an electrolyte containing 0.8 M CuSO_4_, 100 g/L H_2_SO_4_, 0.1 mL/L HCl, and an additive (Chemleader Corporation, Hsinchu, Taiwan). Chemical mechanical planarization (CMP) was then performed to reduce the surface roughness (*R*_q_) to ~3 nm (Figure 1). The 8-inch wafers were cut into various top and bottom dies with sizes of 6 × 6 mm^2^ and 15 × 15 mm^2^, respectively. To remove oxides on the surface of the Cu joints, the dies were cleaned in a citric acid for 30 s, rinsed with deionized (DI) water and then dried with a N_2_ purge. We employed chip-to-die bonders (CA-2000VA, Bondtech Co., Ltd., Kyoto, Japan) to manufacture various direct-bonded chips. The schematic diagram of fabrication processes is shown in Figure 2. Detailed processes can be referred to in our previous study [12]. The cross-sections of the joints were then characterized using focused ion beam (DB-FIB, Field Electron and Ion Company, Hillsboro, OR, USA), and the surface roughness (*R*_q_) was examined by an atomic force microscope (AFM, Bruker Dimension Edge and Dimension Icon, Bruker Company, Billerica, MA, USA).

**Figure 3:**
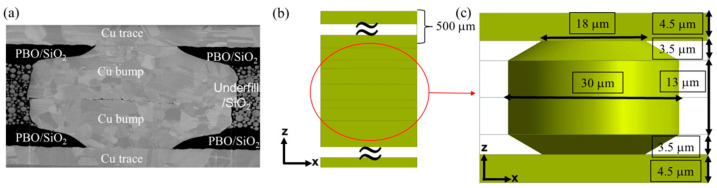
(**a**) Typical cross-sectional FIB image of the Cu-Cu joints. (**b**) The established FEM model. (**c**) Detailed geometry of the 3D bump models.

A commercial finite element method (FEM) code (ANSYS Workbench, Pennsylvania, USA) was used to analyze stress distribution in the Cu joints during thermal cycling tests. We established the FEM models resembling the as-fabricated samples. Figure 3a shows the typically cross-sectional FIB image of the Cu-Cu joints. Herein, two different structures of dielectrics are proposed. One is PBO/UF(U8410-314A)/PBO and the other is pure SiO_2_, as shown in Figure 3a. Note that PBO is a common filling material for advanced packaging. The PBO (HD-8820) used in this study has a high melting point and Young’s modulus. U8410-314A (NAMIC cooperation, Niigata City, Japan) is a filling polymer with a thermal expansion coefficient of ~110 ppm/°C at 125 °C. Figure 3b shows the full 3D FEM model, consisting of two thick Si chips (with a height of 500 μm) and a microbump at the center. The cross-section of the 3D FEM microbump with detailed geometry is shown in Figure 3c. The heights of the microbump, U8410-314A/SiO_2_, and PBO/SiO_2_ were 20, 13, and 3.5 μm, respectively. The diameters of the bonding area and opening (Cu RDL/bump) were correspondingly 30 and 18 μm. The material properties used in the FEM models are listed in Table 1. The boundary conditions were set at the bottom of the solid model. To save computing time, the model planes along *x* and *y* axes were set as symmetry. To ensure the convergence of the numerical analysis, the mesh of each model was refined from 33,197 to 363,514 elements using Solid186 and Solid187. The correlation of temperature and Young’s modulus [23] can be expressed as:

(1) $$E_{Y}=E_{0}-ATexp\left(-\frac{T_{0}}{T}\right)$$

where *E*_0_ (GPa) is the Young’s modulus at 0 K, *A* is the independent constant of temperature, *T*_0_ is the characteristic parameter. *T* is temperature (K).

## 3. Results and Discussion

**Figure 4:**
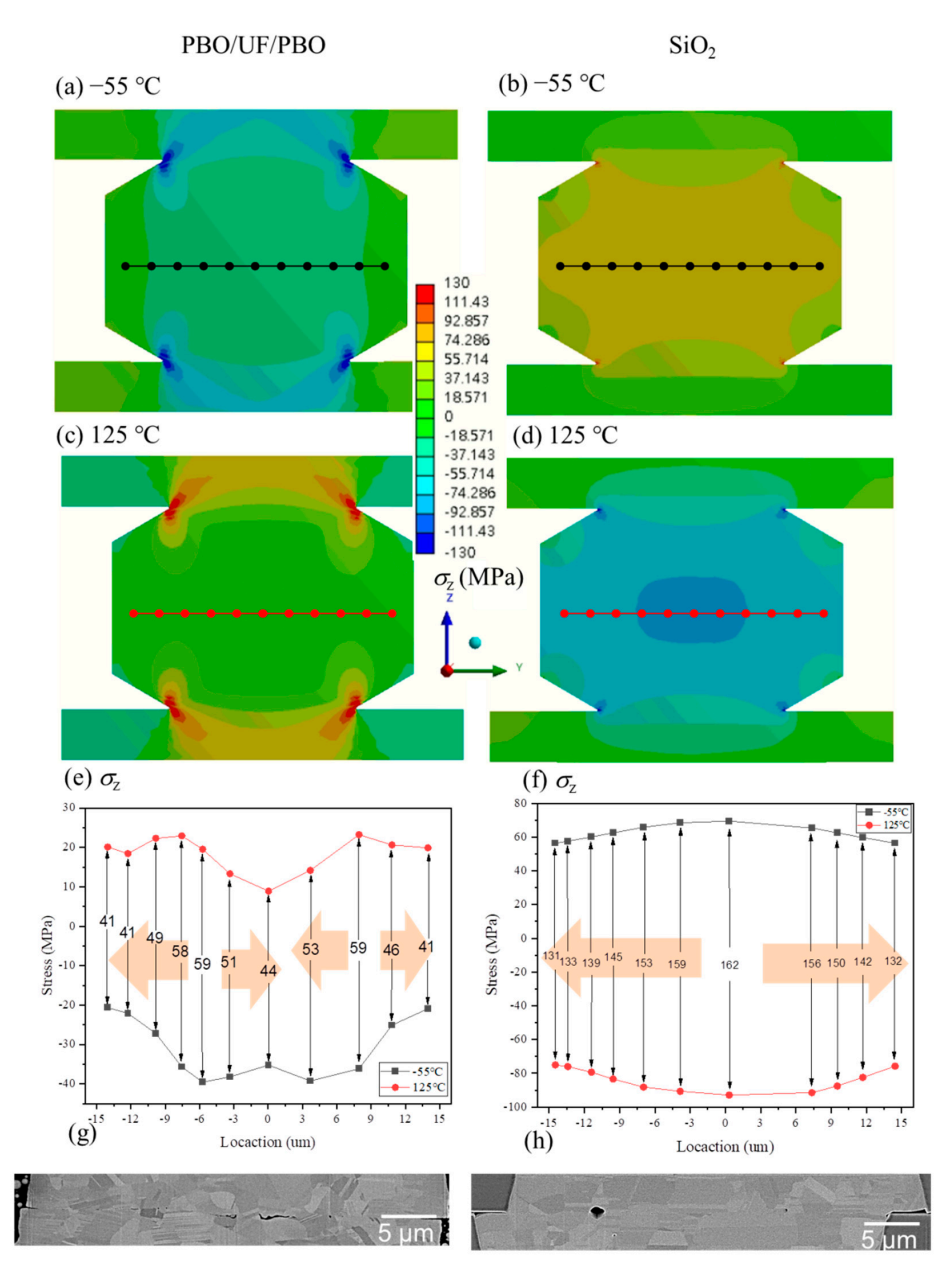
Stress distribution (*σ*_ζ_) in the 3D FEA joints at temperatures −55 °C and 125 °C with two different dielectric structures: PBO/UF/PBO (**a**,**c**,**e**) and SiO_2_ (**b**,**d**,**f**), respectively; (**g**,**h**) are the corresponding FIB images of the joints after the thermal cycling tests.

Under thermal cycling, cracks commonly initiate from pre-existing defects and propagate along the bonding interface and/or grain boundaries. Thus, we constructed the 3D FEM models to address such failure mechanisms. Figure 4 shows the normal stress (*σ*_z_) distribution in the *z*-direction (the *y*-*z* plane) of the FEM joints at −55 °C and 125 °C. We investigated two dielectric structures: case 1 (PBO/UF/PBO) and case 2 (pure SiO_2_). It can be observed that the differences in mechanical and thermal properties resulted in the variations in stress distribution. For case 1, the bonding interface was under compression and tension at −55 °C and 125 °C, respectively. In contrast, in case 2, tensile and compressive stresses were induced in the bonding interface. We plotted the stress values at six points along with the bonding interfaces, as shown in Figure 4e–f. Under thermal cycling, the greatest stress (Δ*σ*_z_) changes (59 MPa) were found at the points of ~8.0 and 5.8 µm from the bump center. They led to significant stress gradients (yellowish arrows) in the microbump. In Figure 4f, the greatest stress (Δ*σ*_z_) change (162 MPa) was found at the bump center. The stress changes gradually decreased along the bonding interface. Figure 4g,h shows the cross-sectional FIB images of the joints after thermal cycling tests. It can be observed that various voids formed at the bonding interfaces. Their locations were in accordance with the directions of the stress gradients. Voids migrated from the higher toward the lower of the stress changes.

Previously, Wu et al. [28] found that voids tend to move and accumulate at the tip of the hole in the interface with a low surface energy. Gondcharton et al. [13] also reported that voids migrate from a high-stress gradient to a low-stress one at a triple grain boundary. In this study, we found that, with different dielectrics, the stress distribution in the Cu joints under thermal cycling was also different. In case 1, stresses concentrated at the two locations (Figure 4f) leading to the accumulation of voids. In case 2, the locations of voids were in the vicinity of the bump edges as a result of stress gradients.

**Figure 5:**
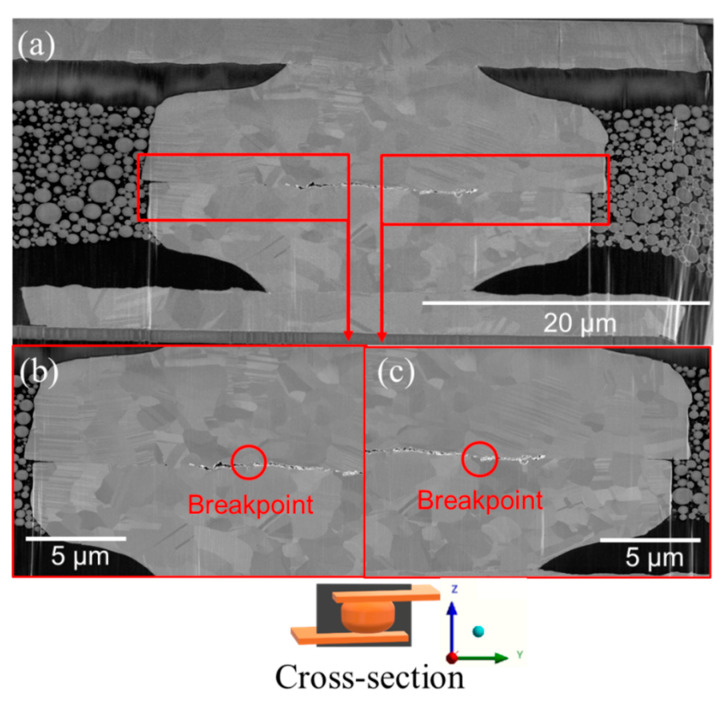
(**a**) Cross-sectional FIB image of the joints after 1000 thermal cycles at a high cycling temperature (300 °C). (**b**,**c**) The enlarged images of (**a**) showing various voids and breakpoints in the bonding interface.

**Figure 6:**
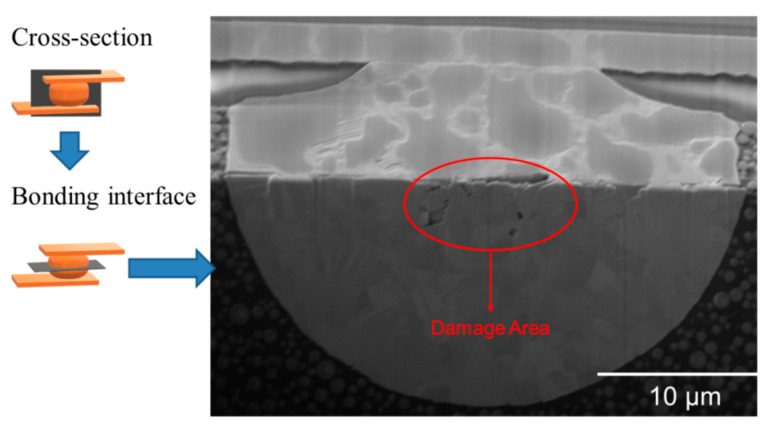
Typical FIB image of the joints bonded at 300 °C/90MPa/30 s prior to thermal cycling tests. Various cracks and/or voids pre-existed in the central region. Under thermal cycling, they tend move from the high stress regions toward the lower stress vicinities.

In addition, we tested the joints at a higher cycling temperature. The cross-sectional FIB image of the joints tested at 300 °C is shown in Figure 5. A large gap was detected at the center of the joints. Various voids formed and aggregated from the center to the edges. The breakpoints appeared due to significant stress changes under thermal cycling. Figure 6 shows the typical FIB image of the joints bonded at 300 °C/90 MPa/30 s prior to thermal cycling tests. It can be seen that various voids pre-existed in the central region. These defects were inevitable during the bonding process. Under thermal cycling, they tend to move toward low stress regions.

**Figure 7:**
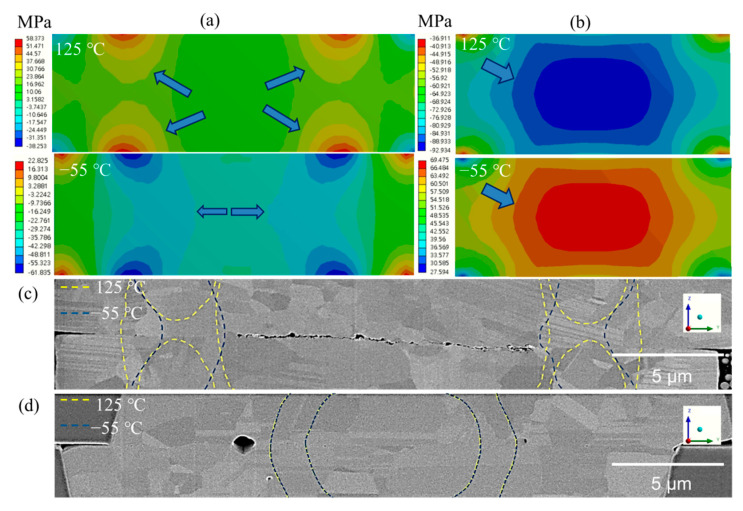
Comparison of numerical and experimental observations for two different dielectric structures: (**a**) PBO/UF/PBO and (**b**) SiO_2_. The yellow and blue dotted lines in the FIB images ((**c**), PBO/UF/PBO) and (**d**), SiO_2_) represent the stress gradient lines at 125 °C and −55 °C, respectively.

**Figure 8:**
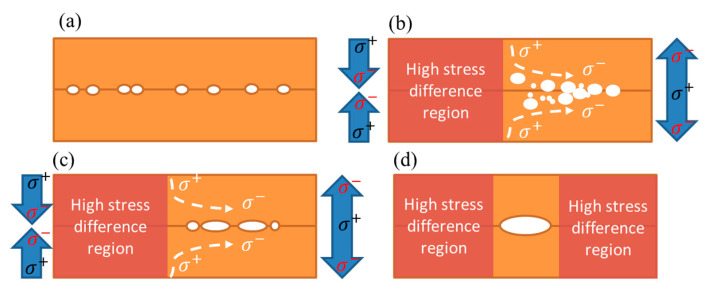
Movement mechanism of interfacial voids in the joints. Various voids pre-exist at the bonding interface of the as-bonded joints (**a**). Under thermal cycling, these voids tend to migrate toward the low stress regions (**b**). Voids merge together until the void density reaches a steady state (**c**). A large void forms between two high stress regions (**d**).

A visual comparison between the simulation and experimental results is shown in Figure 7. In Figure 7a, tensile stresses were located at the two separated regions of the joints with the PBO/UF/PBO structure. The stress distribution at different temperatures was varied. The center region of the joints was not subjected to a significant stress change. The stress distribution in the joints with SiO_2_ is shown in Figure 7b. High tensile stress tended to concentrate at the center. We found that voids were likely to form and expand at the regions with a small variation of stress. Those regions were thus the weakest locations of the bonding. Additionally, stress triaxiality was produced and crack-like voids were generated. Such voids could cause fractures in the central region [29]. The arrows in Figure 7a,b show the regions affected by the stress gradient change. The yellow dotted line shows the stress distribution at 125 °C, and the blue line shows the stress distribution at −55 °C. It was found that only the regions subjected to significant changes on both sides overlapped. The central area was less affected by stress changes. Thus, voids were likely to accumulate in this area (Figure 7c). When the filling dielectric material was replaced by SiO_2_, the stress change was more stable (Figure 7d). No obvious change in the affected area was found. Therefore, voids would migrate to the outside of the joints, reducing the probability of void accumulation in the central area. The mechanism of void accumulation is shown in Figure 8. The stress gradient induced by thermal cycling manipulates the location of void accumulation. Voids are forced to move from high stress regions toward the lower ones (Figure 8b). These voids tend to merge together and slowly reach a steady state of void density (Figure 8c). As these voids are located between two high stress regions, they are under compression and merge to form a larger void (Figure 8c). Such high stress variations are correlated with the changes in the dielectric structures.

In 3D IC packaging, many failures are related to dielectric materials. When high currents are applied to electronic devices to achieve high power operation, the accompanying thermo-mechanical effect can cause cracks at the interfaces between UFs and circuits. Those failures are relatively inconspicuous [30]. They can be attributed to the mismatch in coefficient of thermal expansion (CTE) between different materials. Additionally, cracks are also formed by the accumulation of voids. As they are coated with different dielectric materials, voids may nucleate where hydrostatic tensile stresses are concentrated to reduce free energy [31]. Under thermal cycling, they merge with their neighboring voids to form various clusters. When stresses are not uniformly distributed, the hydrostatic stress gradient will drive these void clusters to form a fragile structure. As listed in Table 1, the UF, PBO, and SiO_2_ used have different CTE values. During the temperature cycle tests, the stresses in the Cu joints were squeezed by the external filler. An M-shaped stress distribution was found (Figure 4e). When replaced by SiO_2_ with a low CTE value, the stress gradient was evenly dispersed to the center of the joints (Figure 4f). Such changes in stress distribution using different dielectrics thus resulted in changes in the accumulated void regions.

## 4. Conclusions

In summary, failure mechanisms in the Cu-Cu joints with different dielectrics under thermal cycling were investigated. The stress distribution in the joints was analyzed and correlated with the experimental observations. We found that interfacial voids were concentrated in specific regions corresponding to the dielectric structures (PBO/UF/PBO and SiO_2_). Under thermal cycling, stress gradients were induced in the joints. Voids were forced to move forward from the high stress locations to the low stress regions. They accumulated and merged with their neighboring voids to reach a steady state. We found that the stress distribution in the joints was in accordance with the dielectric structures leading to the changes in location of accumulated voids. Using the PBO/UF/PBO structure, various defects accumulated in the center of the Cu joints under long-term thermal cycling. This caused the Cu joints to be prone to damage, resulting in their shorter lifetimes. To prevent defect formation, it is recommended to choose SiO_2_ because it has a smaller CTE mismatch with Cu.

## Figures and Tables

**Table 1:** Material properties used in the FEM models [24,25,26,27].

Materials	CTE (ppm/°C)	Thermal Conductivity (W/m·K)	Young’s Modulus (Pa)	Poisson’s Ratio
Cu	16.8	413 (at −73 °C) 401 (at 0 °C) 392 (at 127 °C)	1.1 × 10^11^	0.34
PBO (HD-8820)	64.0	0.2	2.3 × 10^3^	0.30
UF (U8410-314A)	3.0 × 10^1^ (<115 °C) 1.1 × 10^2^ (130 °C)	0.4	8.5 × 10^9^ (<115 °C) 2.0 × 10^8^ (130 °C)	0.30
SiO_2_	2.7	1.3	6.5 × 10^10^	0.28

## Data Availability

Not applicable.
